# Factors associated with persistence or recovery from long COVID 6 months post-SARS-CoV-2 infection

**DOI:** 10.1017/S0950268825100551

**Published:** 2025-09-18

**Authors:** Mulu Woldegiorgis, Lauren Bloomfield, Rosemary Korda, Gemma Cadby, Sera Ngeh, Paul Knight, Andrew Jardine, Jelena Maticevic, Paul Armstrong, Paul Effler

**Affiliations:** 1 National Centre for Epidemiology and Population Health, Australian National University, Canberra, ACT, Australia; 2 Communicable Disease Control Directorate, Western Australia Department of Health, Perth, WA, Australia; 3 School of Medicine, The University of Notre Dame Australia, Fremantle, WA, Australia; 4 Environmental Health Directorate, Western Australia Department of Health, Perth, WA, Australia; 5 Medical School, Pathology & Laboratory Medicine, The University of Western Australia, Perth, WA, Australia

**Keywords:** long COVID, persistent long COVID, long COVID symptoms, follow-up study, health service use, return to work or study, Australia

## Abstract

There are limited data on the illness trajectory for individuals with long COVID. We prospectively followed 1,234 individuals with long COVID at 3 months post-SARS-CoV-2 infection to identify factors associated with persistence or recovery. At 6 months post-infection, 724 (58.7%) had persistent long COVID and 510 (41.3%) had fully recovered. In multivariable analyses, pre-existing health conditions at the time of initial SARS-CoV-2 infection and reporting fatigue, shortness of breath, and cough 3 months post-infection were independent predictors of persistent long COVID. Age, sex, and number of COVID vaccinations were not significantly associated with persistent long COVID. For persons with persistent long COVID, the median number of symptoms remained stable over follow-up, indicating that there had been little symptomatic improvement. A third of those with persistent long COVID reported seeking medical care for their symptoms and a third had ceased or reduced their hours of work/study. Our findings suggest that there may be distinct clinical trajectories for long COVID observed between 3- and 6-month follow-up, that is, persons who experience full recovery and those with minimal clinical improvement, and this may have implications for management of affected individuals.

## Introduction

Long COVID has been characterized as the continuation or development of new symptoms 3 months after the initial SARS-CoV-2 infection, with no other explanation [1]. Approximately 10–30% of persons infected with SARS-CoV-2 develop long COVID. The wide range in estimates of the prevalence of long COVID can be attributed to a number of factors, including a lack of standardized biomarkers and differences in study design, recruitment strategies, symptom assessment tools employed, and health care environments [2, 3]. Although the exact numbers of those living with the condition are uncertain, at least 65 million people are estimated to be experiencing long COVID globally [4, 5].

Long COVID symptoms may be reported months or even years after infection [6–8]. The persistence of long COVID symptoms also leads to ongoing demands on healthcare services and can have substantial negative impacts on the ability to work, resulting in significant economic consequences for individuals and society [9–12].

Because a specific diagnostic test is lacking, there is a reliance on self-reported symptoms to diagnose long COVID [13–15]. The diverse symptomatology associated with this condition, however, makes developing a precise case definition difficult [16, 17]. Even more challenging has been identification of key factors which can explain the varying clinical course of COVID over time [18–20]. Early investigations revealed that symptoms associated with long COVID could increase, decrease, or plateau during the first year following SARS-CoV-2 infection [6, 21]. Several clinical trajectories for individuals with long COVID have recently been identified including cohorts with rapidly decreasing versus highly persistent symptomatology, with the latter group likely responsible for the significant ongoing burden of long COVID from a healthcare and economic perspective [19, 22].

The main aim of this study was to investigate factors associated with persistence of symptoms among individuals who had long COVID 3 months after a first SARS-CoV-2 infection with the Omicron variant in Western Australia (WA).

## Methods

This longitudinal study prospectively followed persons who had long COVID 3 months after SARS-CoV-2 infection out to 6 months. Persons 18 years of age or over with laboratory-confirmed infection between 16 July and 03 August 2022, and long COVID diagnosed in a previous study, were eligible for inclusion [12].

In the earlier study, long COVID was defined as ‘the continuation or development of new symptoms 3 months after the initial SARS-CoV-2 infection with no other explanation’. In this follow-up study, ‘persistent long COVID’ is defined as the presence of one or more COVID-19 illness-related symptom/s or health issue/s 6 months post-SARS-CoV-2 infection among persons who had long COVID 3 months post-infection; ‘recovered long COVID’ is defined as persons who had long COVID at 3 months post-infection who no longer report related symptoms 6 months post-infection. ‘Fully vaccinated’ was defined as receipt of 3 or more doses of a COVID-19 vaccine at least 7 days before SARS-CoV-2 infection, and ‘not fully vaccinated’ was two or fewer doses [12].

In January 2023, the WA Department of Health sent a follow-up survey via text message to persons with long COVID at 3 months who had consented to further follow-up. The responses from the 6-month survey were linked to those from the 3-month survey and information recorded during an interview conducted at the time of their initial SARS-CoV-2 infection, which included demographics, COVID-19 vaccination status prior to infection, and pre-existing health conditions.

Like the 3-month survey, the 6-month survey solicited information on the presence of 22 specific symptoms (henceforth called ‘solicited symptoms’), as well as healthcare utilization and impacts on work or study. Non-respondents were reminded of the opportunity to participate in the research via a text message. Persons who reported having a SARS-CoV-2 re-infection diagnosed by polymerase chain reaction or rapid antigen tests between the 3- and 6-month surveys were excluded.

### Analysis

We compared baseline demographic and health characteristics in people who completed and people who did not complete (did not consent/consented but did not complete) the survey using chi-squared tests.

Among those who completed the 6-month survey, we calculated the number and proportion with persistent long COVID. We described long COVID symptoms reported at 3 months post-infection separately for those with and without persistent long COVID, including the mean number of symptoms and proportions with each of the solicited symptoms.

We used Poisson regression with robust error variance to estimate relative risks (RR), with 95% confidence intervals (CI) of potential predictors of persistent long COVID. To examine the association of symptoms reported at 3 months with persistent long COVID at 6 months, we assessed each symptom individually while treating sex, 10-year age group, region (metropolitan vs. rural), vaccination status, and any significant or long-standing health issues at the time of the acute SARS-CoV-2 infection as potential confounders, using inverse probability weighting (IPW). These potential confounders were selected for inclusion based on our previous work, which found they were associated with long COVID 3 months post-SARS-CoV-2 infection [12]. To quantify the risk of persistent long COVID associated with increased overall symptomatology, we used a multivariable model, simultaneously adjusting for potential confounders and the number of solicited long COVID symptoms reported by participants at 3 months.

To examine health trajectories among those with persistent long COVID, we assessed the change in the number and type of symptoms individuals reported at 3 and 6 months post-infection as well as reductions in work or study and health care visits in the month prior to the survey. Statistical analysis was conducted in Stata 15 [23].

## Results

### Survey response

**Figure 1.**
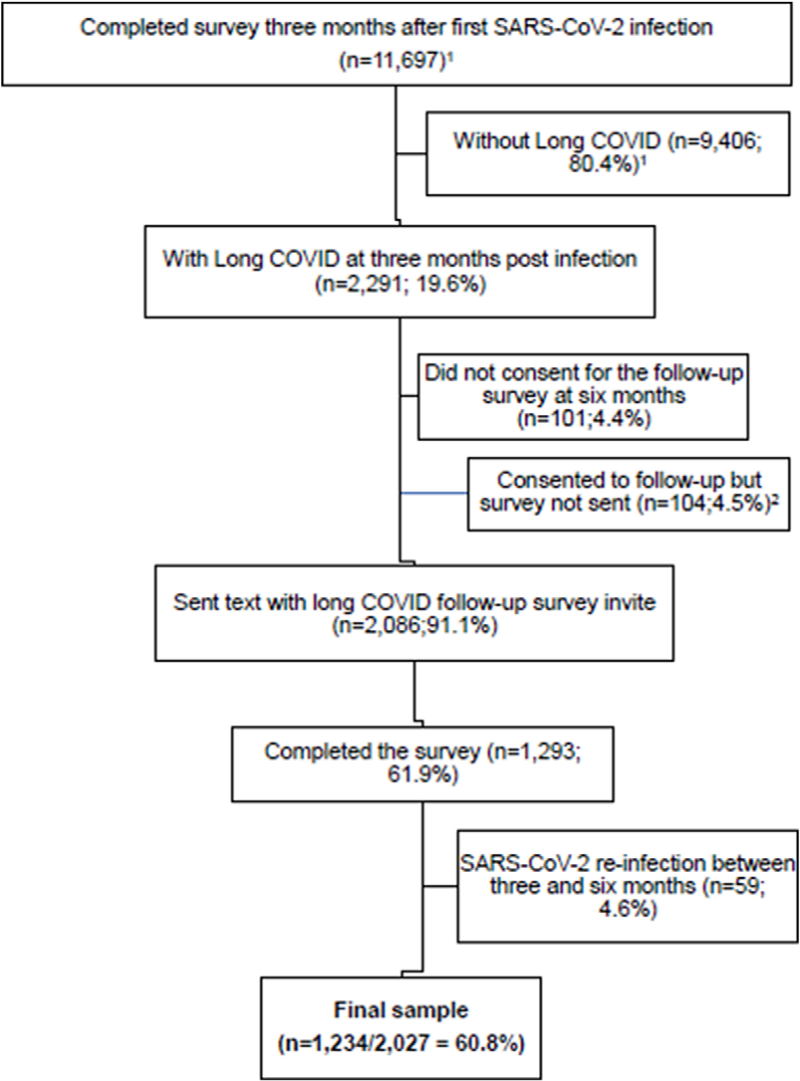
Flowchart of participants for the long COVID follow-up study, Western Australia, 12-30 January 2023. *Notes:* ^1^A total of 11,697 participants completed the three-month survey during the initial study. Of those, 2,291 met the case definition for long COVID three months post-infection. ^2^Survey not sent due to an administrative error.

Of 2,291 participants with long COVID 3 months post-infection, 2,086 (91.1%) consented to follow-up and were sent a text message with a link to the 6-month survey. Of those invited, 1,293 (61.9%) completed the survey. Data for 59 respondents were excluded because re-infection between the 3- and 6-month surveys was retrospectively identified, resulting in a final study population of 1,234 respondents, that is, 60.8% of the 2,027 persons with a single SARS-CoV-2 infection invited to participate (Figure 1).

Characteristics of those who did not consent to follow-up, and those who consented and completed or did not complete the survey, are shown in Table 1. In general, these three groups were similar, with the exception that survey respondents tended to be older and had received more COVID-19 vaccine doses. Females comprised about two-thirds of all three groups (range: 65.8%–67.4%). Importantly, persons who consented to follow-up and completed the 6-month survey were similar to those who consented but did not complete the survey in terms of the mean number of long COVID symptoms reported at 3 months and the proportion reporting any significant or long-standing health issues prior to their initial SARS-CoV-2 infection.

**Table 1.** Baseline characteristics of respondents and non-respondents

Variable	Did not consent for 6-month survey (*n* = 101)		Consented but did not complete the 6-month survey(*n* = 793)		Completed 6-month survey (final study sample) (*n* = 1,234)	
	Number	% (95% CI)	Number	% (95% CI)	Number^1^	% (95% CI)
Sex						
Female	67	67.0 (57.1–75.6)	520	65.8 (64.7–68.8)	828	67.4 (64.7–70.0)
Male	33	33.0 (24.4–43)	270	34.2 (31.2–35.3)	401	42.6 (30.1–35.3)
Age group						
18–29	23	22.8 (15.5–32.1)	115	14.5 (12.1–17.1)	74	6.0 (4.7–7.5)
30–39	22	21.8 (14.7–31.1)	153	19.3 (16.6–22.2)	140	11.4 (9.6–13.2)
40–49	15	14.9 (9.1–23.4)	163	20.6 (17.8–23.5)	214	17.3 (15.3–19.6)
50–59	22	21.8 (14.7–31.1)	195	24.6 (21.6–27.7)	351	28.4 (25.9–31.1)
60–69	16	15.8 (9.9–24.5)	114	14.4 (12–17)	303	24.6 (22.2–27.1)
70 or above	3	3.0 (0.9–9.0)	53	6.7 (5–8.7)	152	12.3 (10.5–14.3)
Region						
Metropolitan	81	80.2 (71.1–87.0)	648	81.7 (78.8–84.3)	999	81.0 (78.7–83.1)
Regional/remote	20	19.8 (13.1–28.9)	145	18.3 (15.7–21.2)	235	19.0 (16.9–21.3)
Number of doses before SARS-CoV–2 infection						
0–2 doses	10	9.9 (5.4–17.6)	57	7.2 (5.5–9.2)	63	5.1 (3.9–6.5)
3 doses	84	83.2 (74.4–89.4)	640	80.7 (77.8–83.4)	867	70.3 (67.6–72.8)
4 or more doses	7	6.9 (3.3–14.0)	96	12.1 (9.9–14.6)	304	24.6 (22.3–27.1)
Self-rated health at 3 months						
Excellent	2	2.0 (0.5–7.7)	12	1.5 (0.8–2.6)	14	1.1 (0.6–1.9)
Good	39	38.6 (29.5–48.6)	321	40.5 (37.0–44.0)	455	36.9 (34.2–39.6)
Fair	47	46.5 (36.9–56.4)	388	48.9 (45.4–52.5)	624	50.6 (47.7–53.4)
Poor	12	11.9 (6.8–19.9)	62	7.8 (6–9.9)	123	10.0 (8.4–11.8)
Not sure	1	1.0 (0.1–6.9)	10	1.3 (0.6–2.3)	18	1.5 (0.9–2.3)
Number of Long COVID symptoms 3 months post-infection						
<6	59	58.4 (48.4–67.8)	400	50.4 (47.0–53.9)	580	47.0 (44.2–49.8)
> = 6)	42	41.6 (32.2–51.6)	393	49.6 (46.1–53.0)	654	53.0 (50.2–55.8)
Any significant or long-standing health issues with SARS-CoV–2 infection						
No	73	72.3 (62.6–80.2)	572	72.1 (68.9–75.2)	821	66.5 (63.8–69.2)
Yes	28	27.7 9 (19.8–37.4)	221	27.9 (24.8–31.1)	413	33.5 (30.8–36.2)

1 Excludes five persons for whom sex was not known.

### Risk of persistent long COVID

#### Recovery or persistence of long COVID 6 months post-infection

Of the 1,234 study participants, 724 (58.7%) reported the presence of one or more COVID-19 illness-related symptom/s or health issues 6 months post-SARS-CoV-2 infection and hence were classified as persistent long COVID; 510 respondents (41.3%) reported no longer having COVID-19 illness-related symptoms or health issues 6 months post-SARS-CoV-2 and were classified as recovered long COVID.

#### Comparing long COVID symptoms reported at 3 months between those who recovered and those with persistent long COVID

Participants with persistent long COVID reported a mean number of 7.2 (95% CI, 6.9–7.6; range 1–20) solicited symptoms 3 months post-infection compared to a mean of 5.3 (95% CI, 4.9–5.6; range 1–19) among those who recovered.

**Figure 2.**
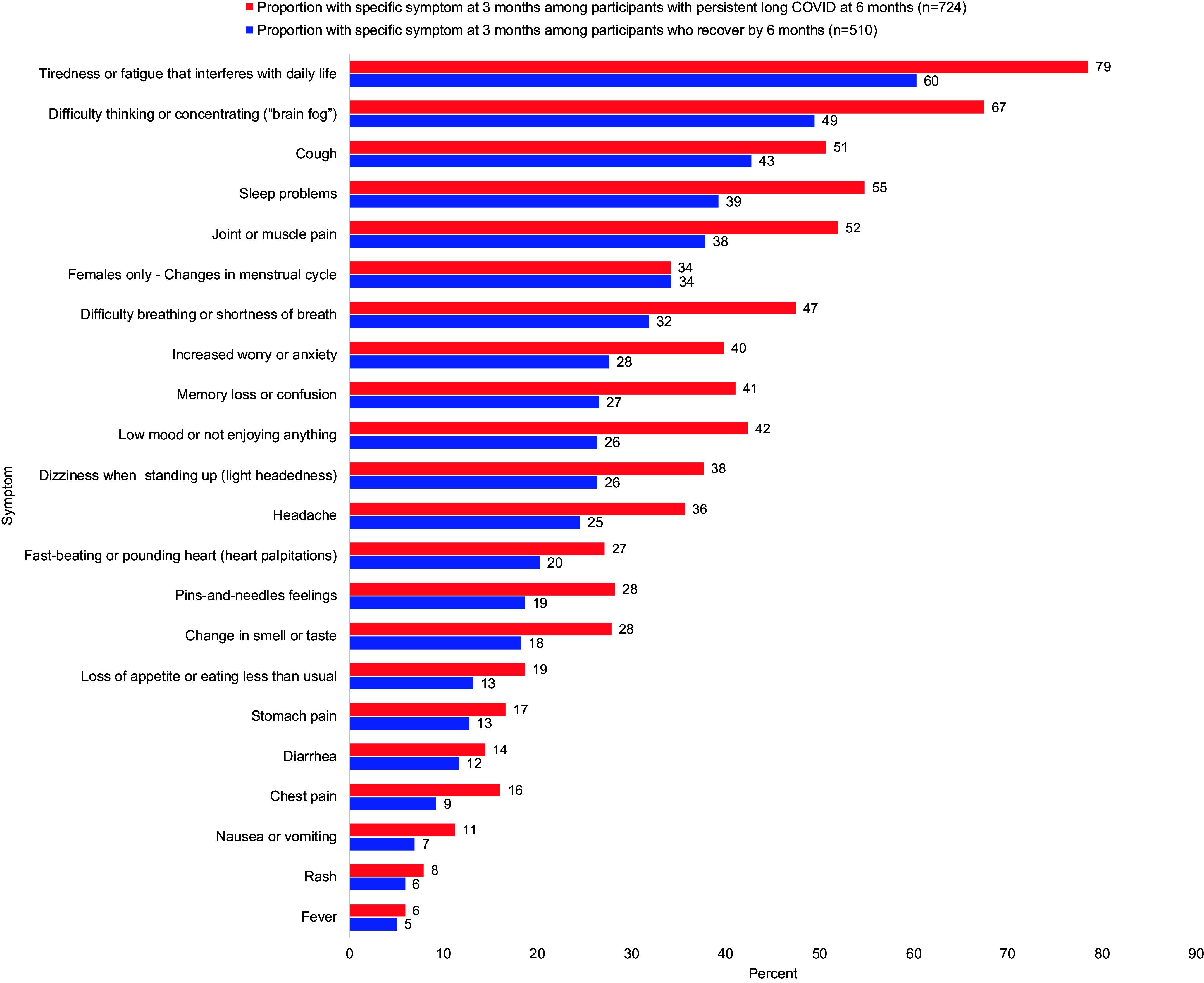
Proportion of respondents reporting each solicited symptom at three months, by recovered vs persistent long COVID.

There was broad similarity in the pattern of solicited symptoms reported at 3 months post-SARS-CoV-2 infection between the cohort that recovered and those with persistent long COVID (Figure 2). Although the cohort with persistent long COVID had a higher frequency of most solicited symptoms at 3 months, the difference in proportions was usually modest, averaging 9.4% (range 0.9%–18.3%) across the 22 symptoms queried.

#### Predictors of persistent long COVID

Of 22 solicited symptoms, 17 were significant predictors of persistent long COVID when adjusted for age, sex, region, vaccination status, and pre-existing health conditions (Table 2). When adjusted for all other solicited symptoms and confounders simultaneously, only tiredness/fatigue (Relative Risk [RR] = 1.27, 95% CI: 1.11–1.47), shortness of breath (RR = 1.15, 95% CI: 1.04–1.26), and cough (RR = 1.10, 95% CI: 1.01–1.20) remained significant independent predictors of persistent long COVID.

**Table 2.** Long COVID symptoms 3 months post-infection associated with persistent long COVID among 1,234 study participants

Variable	Number (%) reporting symptom at 3 months	Unadjusted RR(95% CI)	Adjusted RR^1^(95% CI)	p-value
Tiredness or fatigue that interferes with daily life	875 (70.9%)	1.49 (1.31–1.70)	1.49 (1.31–1.70)	<0.001
Difficulty thinking or concentrating (‘brain fog’)	740 (60.0%)	1.38 (1.24–1.53)	1.37 (1.23–1.53)	<0.001
Sleep problems	596 (48.3%)	1.29 (1.18–1.42)	1.28 (1.16–1.41)	<0.001
Cough	584 (47.3%)	1.14 (1.04–1.25)	1.13 (1.03–1.24)	0.013
Joint or muscle pain	569 (46.1%)	1.26 (1.15–1.39)	1.23 (1.11–1.35)	<0.001
Difficulty breathing or shortness of breath	505 (40.9%)	1.30 (1.19–1.42)	1.35 (1.21–1.51)	<0.001
Low mood or not enjoying anything	440 (35.7%)	1.32 (1.21–1.45)	1.32 (1.20–1.44)	<0.001
Memory loss or confusion	432 (35.0%)	1.29 (1.18–1.41)	1.26 (1.15–1.39)	<0.001
Increased worry or anxiety	429 (34.8%)	1.24 (1.13–1.36)	1.23 (1.12–1.35)	<0.001
Change in menses^2^	99 (34.1%)	1.00 (0.82–1.22)	1.03 (0.85–1.26)	0.740
Dizziness when rising to stand up (light headedness)	406 (32.9%)	1.23 (1.12–1.35)	1.20 (1.09–1.32)	<0.001
Headache	383 (31.0%)	1.23 (1.12–1.35)	1.21 (1.10–1.34)	<0.001
Fast-beating or pounding heart (heart palpitations)	299 (24.2%)	1.16 (1.05–1.28)	1.15 (1.04–1.28)	0.009
Pins-and-needles feelings	299 (24.2%)	1.23 (1.11–1.35)	1.20 (1.09–1.33)	<0.001
Change in smell or taste	294 (23.8%)	1.23 (1.12–1.35)	1.20 (1.09–1.33)	<0.001
Loss of appetite	202 (16.4%)	1.17 (1.05–1.31)	1.16 (1.03–1.30)	0.015
Stomach pain	185 (15.0%)	1.13 (1.00–1.27)	1.08 (0.95–1.24)	0.236
Diarrhoea	163 (13.2%)	1.10 (0.97–1.25)	1.11 (0.97–1.26)	0.133
Chest pain	163 (13.2%)	1.25 (1.12–1.4)	1.26 (1.13–1.40)	<0.001
Nausea or vomiting	116 (9.4%)	1.21 (1.07–1.38)	1.21 (1.06–1.39)	0.006
Rash	87 (7.1%)	1.13 (0.96–1.32)	1.16 (0.98–1.37)	0.076
Fever	73 (5.9%)	1.00 (0.82–1.22)	0.98 (0.79–1.22)	0.877

1 Applied Inverse Probably weighting (IPW). Confounders used to calculate the IPW include age, sex, region, pe-exiting health issues and vaccine doses.

2 Among women of reproductive age (i.e., aged 18 to <=49 years); the denominator is 290.

After multivariable adjustment for sex, age group, region, and vaccination status, the number of symptoms reported at 3 months (i.e., < 6 vs. ≥6) and pre-existing health conditions at the time of initial SARS-CoV-2 infection were significant independent predictors of persistent long COVID (Table 3) Persons with six or more symptoms had a 42% increased risk (RR = 1.42, 95% CI: 1.29–1.58) and those with pre-existing health conditions had a 18% increased risk ([RR] = 1.18, 95% CI: 1.07–1.29) compared to those without (Table 2). When number of symptoms was treated as a numerical variable, each additional symptom increased the risk of persistent long COVID by 5% ([RR] = 1.05, 95% CI: 1.04–1.06). Age, sex, region of residence, and vaccination status were not independent predictors of persistent long COVID in our model (p > 0.05).

**Table 3.** Demographic and health factors associated with persistent long COVID among 1,234 study participants

Variable	Number (%)	Unadjusted RR (95% CI)	Adjusted RR^1^ (95% CI)	p-value
Sex ^2^				
Female	495/828 (59.8%)	1.07 (0.97–1.9)	1.03 (0.93–1.14)	0.542
Male (reference)	224/401 (55.9%)	–	–	
Age group				
18–29 (reference)	40/74 (54.1%)	–	–	
30–39	76/140 (54.3%)	1.00 (0.77–1.30)	1.03 (0.80–1.33)	0.324
40–49	131/214 (61.2%)	1.13 (0.89–1.43)	1.18 (0.93–1.48)	
50–59	210/351 (59.8%)	1.11 (0.88–1.39)	1.10 (0.88–1.38)	
60–69	170/303 (56.1%)	1.04 (0.82–1.31)	1.06 (0.84–1.33)	
70 or above	97/152 (63.8%)	1.18 (0.93–1.50)	1.20 (0.94–1.53)	
Region				
Metropolitan (reference)	578/999 (57.9%)	–	–	
Regional/remote	146/235 (62.1%)	1.07 (0.96–1.20)	1.05 (0.94–1.18)	0.395
Number of doses before SARS-CoV–2 infection				
Not fully vaccinated (0–2 doses)	42/63 (66.7%)	1.15 (0.94–1.41)	1.09 (0.92–1.30)	0.318
Fully vaccinated (3+ doses reference)	682/1171 (58.2%)	–	–	
Number (SD) of long COVID symptoms at 3 months				
<6 (reference)	275/580 (47.4%)	–	–	
6 or more	449/654 (68.7%)	1.45 (1.31–1.60)	1.42 (1.29–1.58)	**<0.001**
Any significant or long-standing health issues at the time of SARS-CoV–2 infection				
No (reference)	449/821 (54.7%)	–	–	
Yes	275/313 (87.9%)	1.22 (1.11–1.34)	1.18 (1.07–1.29)	**0.001**
Total	724/1234 (58.7%)	–	–	

1 Poisson model adjusted for all other characteristics in this Table.

2 Excludes five persons for whom sex was not known.

### Changes in symptoms, and health service use and return to work/study at 6 months, among individuals with persistent long COVID

#### Change in symptoms between 3 and 6 months

Among those with persistent long COVID, the number of solicited symptoms reported remained stable over time. The mean number of symptoms reported at 3 and 6 months post-SARS-CoV-2 infections was 7.2 (standard deviation [SD] = 4.0) and 7.1 (SD = 4.1), respectively, and the median was 7.0 (interquartile range [IQR] = 4–10) at both timepoints.

**Figure 3.**
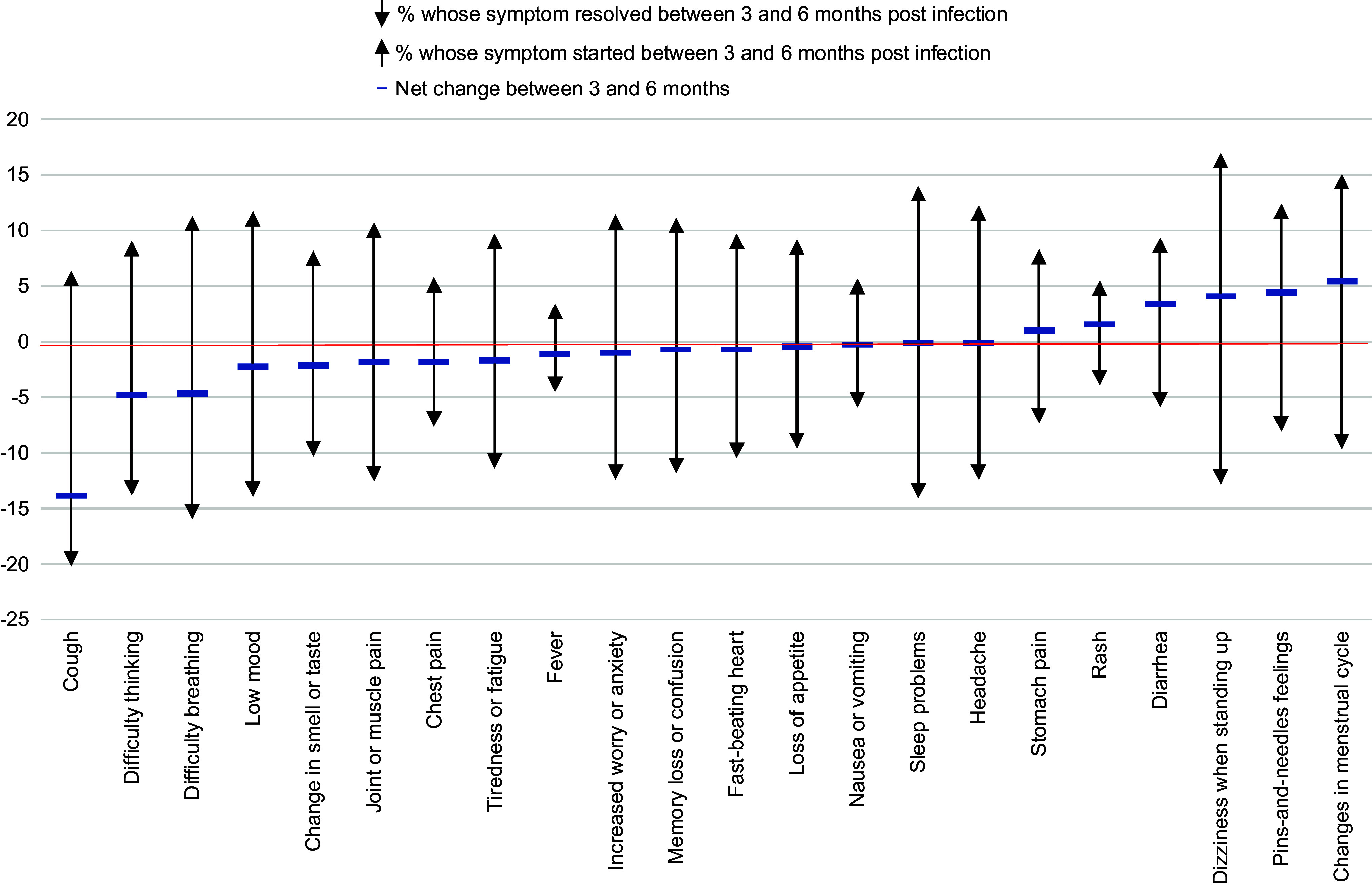
.Proportion of individuals with persistent long COVID reporting resolution or new onset of a solicited symptom at three and six months after SARS-CoV-2 infection

Figure 3 depicts the proportion of individuals who experienced resolution or development of a given solicited symptom across the 3- and 6-month time points. There was variability in the specific symptoms reported by an individual over time. However, the net change in the overall prevalence of a specific solicited symptom between 3 and 6 months was typically modest (< 5%), with the number of individuals who reported the symptom at 3 but not 6 months being largely offset by the number of individuals who reported the symptom at 6 but not 3 months. The only exceptions to this were cough, which saw a net reduction in prevalence of 13.8% and change in menses, with a net increase of 5.3%.

#### Health service use

A third (33.6%; 243/724) of those with persistent long COVID reported accessing health services in the month prior to the 6-month survey due to ongoing symptoms of long COVID. This figure was only marginally lower than the percentage (38.7%) accessing health services in the month prior to the 3-month survey [12]. The vast majority of the healthcare encounters for those with persistent long COVID were outpatient consults with a General Practitioner (97.1%; 236/243); emergency department visits (10.3%; 25/243) and hospital admissions were uncommon (5.8%; 14/243).

#### Return to work or study

Among the 559 persons with persistent long COVID who reported working/studying prior to their SARS-CoV-2 infection, 179 (32.0%) had ceased or reduced their hours of work or study due to long COVID at 6 months of follow-up. This figure was higher than the proportion (17.8%) who reported reducing or discontinuing work or study in the 3-month survey [12]. Of those with persistent long COVID who reported discontinuing or reducing their work or study hours, 68 (38.0%) had already done so at 3 months of follow-up, but 92 (51.4%) reported fully returning to work or study within 3 months of their initial SARS-CoV-2 illness and then subsequently stopping or reducing their hours at 6 months of follow-up.

## Discussion

This follow-up study of persons who had long COVID 3 months post-SARS-CoV-2 infection found that four out of 10 persons reported full recovery from long COVID between 3 and 6 months. Reporting one or more long-standing health issues at the time of the initial SARS-CoV-2 infection and six or more symptoms at 3 months were independent predictors of having persistent long COVID. Examining specific symptoms showed the risk of persistent long COVID was higher for those who reported fatigue, shortness of breath, or cough 3 months post-infection, though for each of these, the attributable increase in risk was modest (i.e., 10–27%).

Importantly, we identified a substantial overlap in terms of the number of solicited symptoms reported at the 3-month time point between the cohort that recovered (mean = 5.3; range 1–19) and those who did not (mean = 7.2; range 1–20). In addition, we observed that the types and frequencies of symptoms reported at 3 months among those with persistent long COVID paralleled, albeit at a higher rate, the symptoms reported among those had who recovered.

Prior to analysing our data, we had anticipated that almost all persons would have some resolution of their symptoms over time and that developing new symptoms more than 3 months after infection would be infrequent. Instead, we found substantial variability in the specific symptoms individuals reported at 3- and 6-month follow-up. Though not anticipated, this observation is not unique to our study population, as considerable heterogeneity in an individual’s long COVID symptomatology over extended follow-up has been reported in other settings [20]. Another unexpected finding was that there was essentially no reduction in the mean number of symptoms reported by those with persistent long COVID across the 3- and 6-month time points, with the median number of solicited symptoms reported remaining at 7.0. This suggests that self-reported clinical improvement was very limited over this time frame.

Precise mechanisms to explain why some persons recover completely from long COVID and others have little improvement in their symptomology over time remain elusive [24]. Our data suggest that the existence of medical issues before SARS-CoV-2 infection may play a role. In addition, fatigue that interferes with daily life reported at 3 months was the symptom most strongly associated with persistent long COVID, followed by dyspnoea. Both symptoms are characteristic of myalgic encephalomyelitis/chronic fatigue syndrome (ME/CFS), as are ‘brain fog’, sleep disruption, and myalgia/arthralgia, which were reported by a majority of persons with persistent long COVID in our study. There is growing evidence that SARS-CoV-2 infection can trigger ME/CFS. Moldofsky et al. report that persistent symptoms experienced by long COVID patients are similar to those described for ME/CFS and a recent meta-analysis found that approximately half of long COVID patients satisfy a diagnosis of ME/CFS [25, 26]. Emerging work suggests that persistence of SARS-CoV-2 infection and development of chronic autonomic dysfunction may be important in explaining ongoing symptomatology associated with long COVID [27–29]. In addition, there is substantial evidence suggesting a role for psychological mechanisms in development of long COVID, but these constructs have been inadequately explored and warrant further investigation [30, 31].

Our study contributes to efforts to better understand the evolution of long COVID illnesses over time. In France, Servier and colleagues identified ‘3 trajectories’ for individuals with long COVID, that is, highly persistent, rapidly decreasing, and slowly decreasing symptoms [19]. However, their study included participants with suspected COVID-19 disease who lacked laboratory evidence of infection, did not report on vaccination status, and spanned a period when three different SARS-CoV-2 variants circulated. Our study extends observations on long COVID illness trajectories to a cohort of highly vaccinated individuals with lab-confirmed infection caused by a single SARS-CoV-2 variant (Omicron), and we observed two distinct trajectories, which ultimately led to full or minimal recovery at 6 months follow-up.

Our findings also underscore the ongoing burden of long COVID on the healthcare system, with a third of those with persistent long COVID seeking medical attention for their symptoms in the month prior to the 6-month survey. Most participants who sought care attended General Practice, highlighting the critical role of primary care in managing long COVID and thus the need to ensure adequate, ongoing resource allocation to primary care settings [12]. In addition, there is emerging evidence to suggest that investment in speciality ‘long COVID clinics’ may be warranted, as early treatment at such clinics has been associated with fewer downstream inpatient stays and reduced mortality [32]. For individuals experiencing long COVID, one of the goals of treatment should be to increase the proportion on a trajectory of rapidly decreasing symptoms. Innovative models of care that can be delivered in primary care settings and focus on patient-led self-care should be prioritized. Given the substantial overlap of symptom clusters between persistent long COVID and ME/CFS, existing ME/CFS care guidelines might be adapted to facilitate standardized diagnosis and management of persistent long COVID [33–35]. The substantive proportion of persons with persistent long COVID in our study who reported symptoms consistent with anxiety and depression suggests that incorporating access to robust mental health services may also be helpful in optimizing recovery [36].

Finally, we found that about a third of those with persistent long COVID were not fully back at work or study 6 months after their initial SARS-CoV-2 infection, including a substantial number of people who had previously fully returned to these activities by 3 months. These data are consistent with other investigations reporting that the impact of the disease on patients’ lives began increasing 6 months after their initial illness and corroborate other studies in documenting the significant economic impact of long COVID for both employees and employers [9, 37–39]. Workers with long COVID face a number of challenges in returning to work, including impairment of cognitive function, decreased physical endurance, mental health issues, and societal stigma [40, 41]. Ultimately, employers can better retain workers experiencing long COVID by creating supportive policies, which, depending on individual circumstances, may include phased return to work and an option for working from home, alterations to hours, duties, and workload, equipment modifications, and time off for healthcare appointments. Occupational health guidelines supporting sustainable return to work for persons with long COVID are available [42–44].

In contrast to some other investigations, our analysis did not identify an association between COVID vaccinations and a reduced risk of persistent long COVID. It must be noted, however, that our study population was not well suited to examine this issue because only 5% of the participants had received fewer than three doses prior to their initial SARS-CoV-2 infection. Given the small size of the unvaccinated comparator group in our study, this result should be interpreted with caution.

This study has several limitations. First, long COVID symptomatology was based on self-reported information, as opposed to clinical observations verified in medical records. This approach is not uncommon for large-scale studies where clinical assessments may not be feasible for all participants and is supported by the fact that, when studied, objective clinical signs of impairment among individuals with persistent long COVID correlate well with self-reported symptoms [45]. Second, despite a reasonably high response rate (60%), there remains a substantial proportion of individuals who did not participate, and it is unknown if the non-respondents had different experiences or outcomes which might affect the results. This concern is mitigated, however, by the fact that respondents were similar to non-respondents in terms of key long COVID symptoms reported at 3 months and the proportion reporting any significant or long-standing health issues prior to SARS-CoV-2 infection, as shown in Table 1. Third, our questionnaire did not capture subjective or objective measures of symptom severity, and this information may be important for assessing clinical predictors of persistent long COVID. Last, our survey did not include questions on some factors associated with long COVID in other settings such socio-economic status, body mass index, ABO blood groups, and smoking [46, 47].

There are, however, several strengths to this research. First, Western Australia’s pandemic experience enabled assessment of long COVID following a single laboratory-proven SARS-CoV-2 infection with the Omicron variant, thus eliminating the potential impact of repeat infections, simultaneous circulation of different variants, and ambiguous timeframes [48]. Second, vaccination status for participants was obtained from a mandatory, population-based national immunization register. Third, we assessed longitudinal data on individuals gathered across three different time points, that is, a detailed case investigation that occurred at the time of their initial SARS-CoV-2 infection and follow-up surveys conducted at 3 and 6 months. This approach reduces the potential for bias and provides an opportunity to examine changes in the symptom profile of long COVID in the same individual over time.

In conclusion, longitudinal follow-up among a cohort of individuals with long COVID 3 months post-infection found the risk of persistent long COVID at 6 months was greater for those with pre-existing health issues at the time of infection and ongoing fatigue and respiratory symptoms at 3 months. Importantly, we observed that many highly symptomatic persons fully recovered by 6 months and that there was substantial overlap in long COVID symptomatology at 3 months among those who recovered and those who did not. Of note, the cohort with persistent long COVID reported almost no improvement in overall symptomatology between the 3- and 6-month timepoints. These data suggest that there may be distinct clinical trajectories for long COVID between 3- and 6-month follow-up, that is, full versus minimal recovery, which could have implications for support and management of affected individuals.

## Data Availability

The data underlying this report are available from the WA Department of Health upon request and approval.

## References

[1] World Health Organization. A Clinical Case Definition of Post COVID-19 Condition by a Delphi Consensus*, 6 October* 2021. COVID-19: Clinical Care 2021 [cited 2022 June 7]; Available from: https://www.who.int/publications/i/item/WHO-2019-nCoV-Post_COVID-19_condition-Clinical_case_definition-2021.1.

[2] Greenhalgh T, et al. (2024) Long COVID: A clinical update. The Lancet 404(10453), 707–724. 10.1016/S0140-6736(24)01136-X 39096925

[3] Alwan NA and Johnson L (2021) Defining long COVID: Going back to the start. The Medicus 2(5), 501–504. 10.1016/j.medj.2021.03.003 PMC7992371 33786465

[4] Lancet T (2023) Long COVID: 3 years in. Lancet 401(10379), 795. 36906338 10.1016/S0140-6736(23)00493-2 PMC9998094

[5] Davis HE, et al. (2023) Long COVID: Major findings, mechanisms and recommendations. Nature Reviews Microbiology 21(3), 133–146. 36639608 10.1038/s41579-022-00846-2 PMC9839201

[6] Davis HE, et al. (2021) Characterizing long COVID in an international cohort: 7 months of symptoms and their impact. EClinicalMedicine 38, 101019. 34308300 10.1016/j.eclinm.2021.101019 PMC8280690

[7] Groff D, et al. (2021) Short-term and long-term rates of postacute sequelae of SARS-CoV-2 infection: A systematic review. JAMA Network Open 4(10), e2128568. 34643720 10.1001/jamanetworkopen.2021.28568 PMC8515212

[8] Oelsner EC, et al. (2024) Epidemiologic features of recovery from SARS-CoV-2 infection. JAMA Network Open 7(6), e2417440. 38884994 10.1001/jamanetworkopen.2024.17440 PMC11184459

[9] Tene L, et al. (2023) Risk factors, health outcomes, healthcare services utilization, and direct medical costs of patients with long COVID. International Journal of Infectious Diseases 128, 3–10. 36529373 10.1016/j.ijid.2022.12.002

[10] Parums DV (2021) Long COVID, or post-COVID syndrome, and the global impact on health care. Medical science monitor: international medical journal of experimental and clinical research 27, e933446–e933441. 34092779 10.12659/MSM.933446 PMC8194290

[11] Macpherson K, et al. (2022) Experiences of living with long COVID and of accessing healthcare services: A qualitative systematic review. BMJ Open 12(1), e050979. 10.1136/bmjopen-2021-050979 PMC8753091 35017239

[12] Woldegiorgis M, et al. (2024) Long COVID in a highly vaccinated but largely unexposed Australian population following the 2022 SARS-CoV-2 omicron wave: A cross-sectional survey. Medical Journal of Australia 220(6), 323–330. 38508863 10.5694/mja2.52256

[13] Wu Y, et al. (2024) Factors associated with long COVID: Insights from two Nationwide surveys. The American Journal of Medicine 137(6), 515–519. 38490304 10.1016/j.amjmed.2024.02.032

[14] Bonsaksen T, et al. (2022) Self-reported long COVID in the general population: Sociodemographic and health correlates in a cross-National Sample. Life (Basel) 12(6). 10.3390/life12060901 PMC9228837 35743932

[15] Krysa JA, et al. (2023) Understanding the experience of long COVID symptoms in hospitalized and non-hospitalized individuals: A random, cross-sectional survey study. Healthcare (Basel) 11(9). 10.3390/healthcare11091309 PMC10178853 37174851

[16] Iwasaki A and Putrino D (2023) Why we need a deeper understanding of the pathophysiology of long COVID. The Lancet Infectious Diseases 23(4), 393–395. 36967698 10.1016/S1473-3099(23)00053-1 PMC9928485

[17] Ely EW, Brown LM and Fineberg HV (2024) Long Covid defined. The New England Journal of Medicine. 10.1056/NEJMsb2408466 PMC11687645 39083764

[18] Buonsenso D and Tantisira KG (2024) Long COVID and SARS-CoV-2 persistence: New answers, more questions. The Lancet Infectious Diseases. 10.1016/S1473-3099(24)00216-0 38663424

[19] Servier C, et al. (2023) Trajectories of the evolution of post-COVID-19 condition, up to two years after symptoms onset. International Journal of Infectious Diseases 133, 67–74. 37182548 10.1016/j.ijid.2023.05.007 PMC10176960

[20] Ballouz T, et al. (2023) Recovery and symptom trajectories up to two years after SARS-CoV-2 infection: Population based, longitudinal cohort study. BMJ 381, e074425. 37257891 10.1136/bmj-2022-074425 PMC10230608

[21] Tran VT, et al. (2022) Course of post COVID-19 disease symptoms over time in the ComPaRe long COVID prospective e-cohort. Nature Communications 13(1), 1812. 10.1038/s41467-022-29513-z PMC8983754 35383197

[22] Fischer A, et al. (2025) Trajectories of persisting Covid-19 symptoms up to 24 months after acute infection: Findings from the Predi-Covid cohort study. BMC Infectious Diseases 25(1), 603. 40281467 10.1186/s12879-025-11023-0 PMC12023393

[23] StataCorp (2017) Stata Statistical Software: Release 18. College Station, TX: StataCorp LLC

[24] Gheorghita R, et al. (2024) The knowns and unknowns of long COVID-19: From mechanisms to therapeutical approaches. Frontiers in Immunology 15, 1344086. 38500880 10.3389/fimmu.2024.1344086 PMC10944866

[25] Moldofsky H and Patcai J (2011) Chronic widespread musculoskeletal pain, fatigue, depression and disordered sleep in chronic post-SARS syndrome; a case-controlled study. BMC Neurology 11, 37. 21435231 10.1186/1471-2377-11-37 PMC3071317

[26] Dehlia A and Guthridge MA (2024) The persistence of myalgic encephalomyelitis/chronic fatigue syndrome (ME/CFS) after SARS-CoV-2 infection: A systematic review and meta-analysis. Journal of Infection 89(6), 106297. 39353473 10.1016/j.jinf.2024.106297

[27] Zuo W, et al. (2024) The persistence of SARS-CoV-2 in tissues and its association with long COVID symptoms: A cross-sectional cohort study in China. The Lancet Infectious Diseases 24(8), 845–855. 38663423 10.1016/S1473-3099(24)00171-3

[28] Giunta S, et al. (2024) Long-COVID-19 autonomic dysfunction: An integrated view in the framework of inflammaging. Mechanisms of Ageing and Development 218, 111915. 38354789 10.1016/j.mad.2024.111915

[29] Fedorowski A and Sutton R (2023) Autonomic dysfunction and postural orthostatic tachycardia syndrome in post-acute COVID-19 syndrome. Nature Reviews Cardiology 20(5), 281–282. 10.1038/s41569-023-00842-w PMC9893964 36732397

[30] Engelmann P, et al. (2024) Psychological factors associated with long COVID: A systematic review and meta-analysis. EClinicalMedicine 74. 10.1016/j.eclinm.2024.102756 PMC11701445 39764180

[31] Lemogne C, et al. (2023) Why the hypothesis of psychological mechanisms in long COVID is worth considering. Journal of Psychosomatic Research 165, 111135. 36623391 10.1016/j.jpsychores.2022.111135 PMC9825049

[32] Gong, K.D., et al., Assessing the Impact of Post-COVID Clinics on 6-Month Health Care Utilization for Patients with Long COVID: A Single-Center Experience. Mayo Clinic Proceedings: Innovations, Quality & Outcomes, 2025. 9(3): p. 100603. 10.1016/j.mayocpiqo.2025.100603 PMC12002763 40248479

[33] Lapp CW (2019) Initiating care of a patient with myalgic encephalomyelitis/chronic fatigue syndrome (ME/CFS). Frontiers in Pediatrics 6, 415. 30740389 10.3389/fped.2018.00415 PMC6357921

[34] Carruthers BM, et al. (2003) Myalgic encephalomyelitis/chronic fatigue syndrome: Clinical working case definition, diagnostic and treatment protocols. Journal of chronic fatigue syndrome 11(1), 7–115.

[35] Cortes Rivera M, et al. (2019) Myalgic encephalomyelitis/chronic fatigue syndrome: A comprehensive review. Diagnostics 9(3), 91. 31394725 10.3390/diagnostics9030091 PMC6787585

[36] Luo S, et al. (2023) An overview of long COVID support services in Australia and international clinical guidelines, with a proposed care model in a global context. Public Health Reviews 44, 1606084. 37811128 10.3389/phrs.2023.1606084 PMC10556237

[37] Reuschke D and Houston D (2023) The impact of long COVID on the UK workforce. Applied Economics Letters 30(18), 2510–2514.

[38] Kohn L, et al. (2024) Long COVID and return to work: A qualitative study. Occupational Medicine 74(1), 29–36. 36480262 10.1093/occmed/kqac119

[39] Gualano MR, et al. (2022) Returning to work and the impact of post COVID-19 condition: A systematic review. Work 73(2), 405–413. 35938280 10.3233/WOR-220103

[40] Clutterbuck D, et al. (2024) Barriers to healthcare access and experiences of stigma: Findings from a coproduced long Covid case-finding study. Health Expectations 27(2), e14037. 38634418 10.1111/hex.14037 PMC11024953

[41] Diversity council Australia. Supporting Employees with Long COVID to Return to Work. 2022 [cited 2025 13 May]; Available from: https://www.dca.org.au/news/blog/supporting-employees-long-covid-return-work.

[42] Stave GM, Nabeel I and Durand-Moreau Q (2023) Long COVID–ACOEM guidance statement. Journal of Occupational and Environmental Medicine. 10.1097/JOM.0000000000003059. 38588073

[43] Howard, J., M. Cloeren, and G. Vanichkachorn. *Long COVID and occupational medicine practice.* 2024 [cited 2025 30 June]; Available from: https://blogs.cdc.gov/niosh-science-blog/2024/01/22/long-covid-om/. 10.1097/JOM.0000000000002961 37696788

[44] Rayner C, Burton K and MacDonald E (2025) Guidelines for a sustainable return to work with long COVID. Occupational Medicine 75(1), 9–13. 40190125 10.1093/occmed/kqae141

[45] Peter RS, et al. (2025) Persistent symptoms and clinical findings in adults with post-acute sequelae of COVID-19/post-COVID-19 syndrome in the second year after acute infection: A population-based, nested case-control study. PLoS Medicine 22(1), e1004511. 39847575 10.1371/journal.pmed.1004511 PMC12005676

[46] Luo D, et al. (2024) Prevalence and risk factors for persistent symptoms after COVID-19: A systematic review and meta-analysis. Clinical Microbiology and Infection 30(3), 328–335. 37866679 10.1016/j.cmi.2023.10.016

[47] Wong MC, et al. (2023) Epidemiology, Symptomatology, and Risk Factors for Long COVID Symptoms: Population-Based, Multicenter Study, Vol. 9. JMIR Public Health Surveill, e42315 10.2196/42315 PMC9994465 36645453

[48] Bloomfield LE, et al. (2023) SARS-CoV-2 vaccine effectiveness against omicron variant in infection-naive population, Australia, 2022. Emerging Infectious Diseases 29(6), 1162. 37141626 10.3201/eid2906.230130 PMC10202853

